# Cortical networks underlying successful control of nociceptive processing using real-time fMRI

**DOI:** 10.3389/fpain.2022.969867

**Published:** 2022-10-24

**Authors:** Maide Bucolo, Mariela Rance, Frauke Nees, Michaela Ruttorf, Giovanna Stella, Nicolò Monarca, Jamila Andoh, Herta Flor

**Affiliations:** ^1^ Department of Electrical Electronic and Computer Engineering, University of Catania, Catania, Italy; ^2^ Institute of Cognitive and Clinical Neuroscience, Central Institute of Mental Health, Medical Faculty Mannheim, Heidelberg University, Mannheim, Germany; ^3^ Institute of Medical Psychology and Sociology, University Medical Center Schleswig-Holstein, Kiel, Germany; ^4^ Computer Assisted Clinical Medicine, Medical Faculty Mannheim, Heidelberg University, Mannheim, Germany; ^5^ Department of Psychiatry and Psychotherapy, Central Institute of Mental Health, Medical Faculty Mannheim, Heidelberg University, Mannheim, Germany

**Keywords:** brain connectivity, regulation of neural activity, nociceptive processing, coherence analysis, fMRI

## Abstract

Real-time fMRI (rt-fMRI) enables self-regulation of neural activity in localized brain regions through neurofeedback. Previous studies showed successful up- and down-regulation of neural activity in the anterior cingulate cortex (ACC) and the insula (Ins) during nociceptive stimulation. Such self-regulation capacity is, however, variable across subjects, possibly related to the ability of cognitive top-down control of pain. Moreover, how specific brain areas interact to enable successful regulation of nociceptive processing and neurofeedback-based brain modulation is not well understood. A connectivity analysis framework in the frequency domain was used to examine the up- or down-regulation in the ACC and Ins and pain intensity and unpleasantness ratings were assessed. We found that successful up- and down-regulation was mediated by the ACC and by its functional connectivity with the Ins and secondary somatosensory cortex. There was no significant relationship between successful up- or downregulation and pain ratings. These findings demonstrate functional interactions between brain areas involved in nociceptive processing during regulation of ACC and Ins activity, and the relevance of the frequency domain connectivity analysis for real-time fMRI. Moreover, despite successful neural regulation, there was no change in pain ratings, suggesting that pain is a complex perception, which may be more difficult to modify than other sensory or emotional processes.

## Introduction

Real time functional magnetic resonance imaging (rt-fMRI) permits the feedback of neuronal activity, which can then be controlled and regulated. rt-fMRI has been well established over the past 15 years (1, 2) and has often been associated with behavioral changes (3), including pain perception (4), although this could not be consistently replicated (5). Brain responses to nociceptive processing have been shown to involve areas such as primary (SI) and secondary (SII) somatosensory cortices, insula (Ins), the anterior (ACC) or the mid-cingulate (MCC) cortices (6, 7).

The prefrontal cortex (PFC) has also been shown to be involved in pain processing but may be more important in chronic than acute pain (8, 9), and therefore may not be an ideal target for neuromodulation. In addition, the PFC structure is quite complex, and includes different regions, namely, dorsal, medial and ventral prefrontal cortices, involved in various aspects of pain processing, e.g., intensity of pain, spatial aspects of pain processing, emotion regulation, but also involved in various cognitive processes such as attention or decision making (10, 11).

The rostral anterior cingulate cortex (rACC) in particular, has been involved in pain regulation (12, 13), and is therefore a target of choice for rt-fMRI studies (4). In a previous study, we showed that participants could successfully downregulate neural activity related to nociceptive processing in the rACC and the posterior insula (pIns) and upregulate pInsL but not rACC (14). Upregulation or downregulation of either region was unrelated to pain intensity or unpleasantness ratings. The ability to successfully regulate brain activity was also shown to be variable across participants (14), which might be related to lack of cognitive top-down control of pain and deserves further investigation.

We also showed that lower covariation between the two regions correlated positively with the training effect and thus learning, suggesting that the state of the network involved in the processing of pain should be considered in the modulation of pain-evoked activation and related behavioral effects (15). Therefore, in this study, we aimed at examining functional connectivity in pInsL and in rACC and their effect on learning.

In addition, it is unclear how brain areas interact to enable successful regulation of nociceptive processing (16) and neurofeedback-based brain modulation (17, 18). For example, Hinterberger et al., analyzed successful regulation of slow cortical potentials and found that a number of brain regions were involved in successful regulation with a focus on sensorimotor and frontal control regions (19).

We aimed to assess the temporal dependence of activation patterns between brain regions, specifically, the functional connectivity of regulation- and pain-associated brain regions during up- or down-regulation of neural activity related to nociceptive processing. The methodological framework used here to evaluate functional connectivity combines signal processing from data-driven mathematical methods and complex network analysis (20). This integrated approach has previously been applied to various brain signals from electroencephalographic (EEG), magnetoencephalographic (MEG) (21, 22), and functional magnetic resonance imaging (fMRI) data (23). Despite the proliferation of mathematical methods and toolboxes (24–26), there is no general consensus on the most robust and efficient way to assess functional connectivity (27). The use of different computational parameters such as the frequency range, time lag, or the choice of a significance threshold can affect the results at an individual and group level analysis (28, 29). Group analyses allow statistical measures on the validity of the result, but are still affected by sample size and individual variability in complex brain activity and can sometimes hide relevant key brain mechanisms. We investigated some of these issues in our previous works (30, 31) and established a connectivity analysis framework in the frequency domain that we used in the present study. We assessed the effect of self-regulation of the activity from two target ROIs, the rACC and pIns and examined functional connectivity to other areas such as the somatosensory cortex (SII), the anterior and posterior insula (aIns, pIns) and MCC. Based on its role in top-down control, we expected the ACC to play a key role in successful regulation of nociceptive processing and to show functional connections to SII, pIns, MCC.

We added the posterior insula, because it has been involved in the sensory pain aspects (32–34) and was associated with a reliable activation pattern across all subjects (14).

## Materials and methods

### Participants

Ten healthy right-handed participants were enrolled in the study [mean age, standard deviation *M* = 29.0, SD = 6.48, range (20, 41)], four females (*M* = 27.0, SD = 3.92), and six males (*M* = 30, SD = 7.81). Exclusion criteria were cardiovascular or neurological disorders, brain injury, acute pain, current analgesic medication, pregnancy, lifetime and current substance abuse or dependence, any mental disorder, and metallic implants. The study adhered to the Declaration of Helsinki and was approved by the Ethics Committee of the Medical Faculty Mannheim, Heidelberg University, Germany. All subjects gave written informed consent after a detailed description of the complete study. The sample of this study is identical to that described in a previous study (14). Here we reanalysed the data with respect to patterns of connectivity in Learners and non-Learners of neurofeedback control.

### fMRI neurofeedback procedure

**Figure 1:**
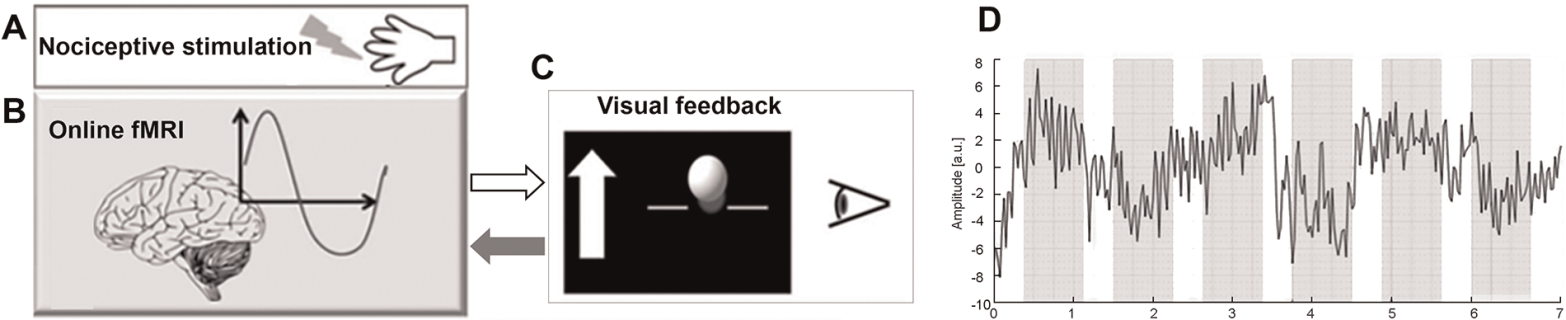
Schema of the fMRI neurofeedback setup. (**A**) Painful electrical stimuli were applied over the fourth digit of the right hand. (**B**) Online pre-processing and statistical analysis of the BOLD response were carried out from the target ROIs (rACC, pInsL) and a control ROI (UNR). (**C**) Differences in BOLD response between target ROIs and UNR ROI were computed and represented by a moving ball in front of a black background on a display screen. On the left side of the ball, a white arrow was displayed with the up or down directions depending on if the participants had to up- or down-regulate neural activity from the target ROIs. (**D**) Trend of a ROI time series in a trial of 7 min where the regulation (grey) and non-regulation (white) phases are highlighted.

The neurofeedback protocol consisted of a baseline run and 24 training trials spread over the course of 4 consecutive days. On the first day, the participants were introduced to the experimental setup and protocol, and the baseline run was recorded. Each session (training day) consisted of six successive training trials; each trial of 7 min was composed of six regulation phases, each lasting 45 s and seven non-regulation phases, each lasting 22.5 s, evenly distributed across each session. The sequence of regulation and non-regulation phases is depicted, the overall duration of a trial is 7 min and consists of 258 samples based on the acquisition time TR = 1.5 s (sampling frequency 0.66 Hz). On the right of Figure 1, as an example, the time-series extracted from a ROI fMRI is shown. During the regulation phases, 2 ms of painful electrical stimulation at a frequency of 2 Hz were carried out using a digitimer DS7A stimulator and applied over the fourth digit of the right hand using concentric bipolar electrodes (see Figure 1A).

Individual detection and pain thresholds were determined by the method of limits, averaging over the last two of three ascending and descending stimulation sequences (15). Pain tolerance was averaged over the last two of three ascending stimulation sequences. Stimulation strength was set at 70% between pain threshold and pain tolerance and adjusted to be rated between 6 and 7 on an 11 point verbal rating scale (ranging from 0 = no pain to 10 = strongest imaginable pain), allowing for a possible increase or decrease of perceived pain strength. The individually adjusted mean stimulation strength was 2.27 mA (SD = 1.76), the pre baseline intensity of this stimulus was rated as 6.40 (SD = 0.61) and the unpleasantness was assessed on a verbal rating scale (raining from 0 = not unpleasant to 10 = extremely unpleasant) amounting to 6.70 (SD = 1.32). The postbaseline stimulus intensity was rated 6.10 (SD = 1.68) and the pain unpleasantness 7.25 (SD = 1.51).

The visual feedback consisted of a moving blue or yellow ball in front of a black background (Figure 1C).

During the regulation phases of the training trials, a stationary white arrow appeared next to the ball on the left side of the screen indicating the vertical direction in which the ball should be moved. Movements of the ball corresponded to changes in the computed BOLD signal from the regions of interest (ROI), i.e., rACC or pInsL and a control ROI (UNR), with activity unrelated to the nociceptive stimulation or pain processing (located in the parietal lobe, bordering the occipital lobe and the height of pInsL), see (14) for detailed information, Figure 1B. The target ROIs, i.e., rACC or pInsL were discernible by the colour of the moving ball (blue or yellow) for rACC and pInsL and the colour was randomized across participants. The baseline run was similar to the training trial, the subjects were presented a stationary white ball on the screen, but no visual feedback was given. The participants were instructed that the vertical change of the blue or yellow ball was an indicator of their own brain activity in selected brain regions and that they would be able to observe the changes with a delay of a few seconds. The subjects were allowed to use any kind of strategy that would not involve body movement (e.g., muscle tension or relaxation). During the non-regulation phases, i.e., in the absence of visual feedback, the participants were told to perform simple mental arithmetic for the purpose of stopping regulation attempts and ensuring comparability across subjects.

### MRI acquisition

MRI data were acquired on a 3 T MAGNETOM Trio TIM whole body scanner using a standard 12-channel head coil (Siemens Medical Solutions, Erlangen, Germany). fMRI data were acquired using gradient-echo and echo-planar imaging (EPI) sequence (TR/TE = 1,500/22 ms, matrix size 96 × 96, flip angle 90◦, and bandwidth BW = 1,270 Hz/px). Twenty-four AC/PC aligned slices were acquired with voxel size 2.2 mm × 2.2 mm × 3.5 mm and 0.5 mm gap. A three-dimensional fast low angle shot high-resolution T1-weighted anatomical scan was also acquired for each participant (TR/TE = 23/5.02 ms, matrix size 448 × 448, flip angle 25◦, BW 190 Hz/px, voxel size 0.5 mm × 0.5 mm × 1.0 mm) as anatomical reference. Foam pegs (Siemens Medical Solutions, Erlangen, Germany) were used to immobilize the subject's head during MR scanning.

### MRI pre-processing and statistical analysis

#### Online fMRI data pre-processing and statistical analyses

**Figure 2:**
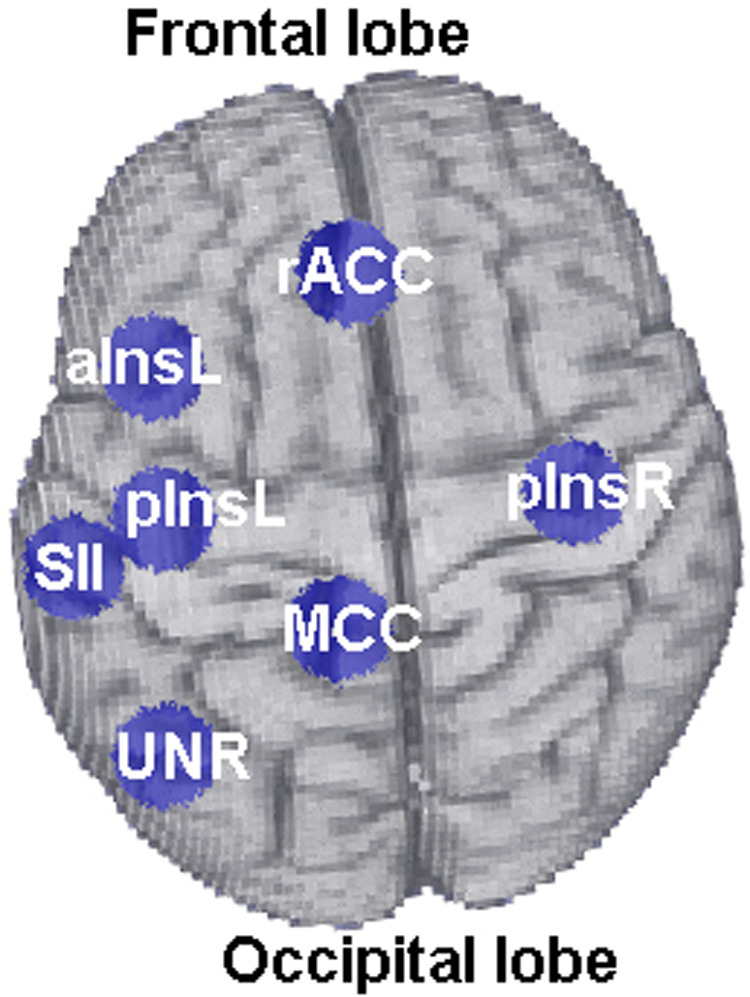
Location of the six ROIs (rACC, pInsL, pInsR, MCC, aInsL, SII) used for functional connectivity analyses. The ROI “UNR” corresponds to the control ROI and is shown for illustration purposes.

Brain responses to nociceptive stimulation were recorded and analysed in real-time during the fMRI acquisition using Turbo BrainVoyager Version 1.1 (Brain Innovation, Maastricht, TheNetherlands) as described in (2). The mean BOLD signal change from two target regions of interest (ROIs), i.e., rACC and pInsL was compared with a control region (“UNR”), see Figure 2 and Table 1 for coordinates. The feedback signal was calculated as the difference of the percent BOLD signal change between one of the target ROIs and the UNR ROI (Figure 1B) and visually fed back to the subject in the form of a moving ball on a screen (Figure 1C). The feedback computation and visualization were performed with in-house written scripts based on Presentation® Version 13.0 Build 01.23.09 (Neurobehavioral Systems Inc., Albany, CA, USA) on another computer connected with Turbo BrainVoyager *via* LAN. The location of the rACC, pInsL and UNR regions was determined in an offline analysis of the baseline run. The criteria for the target ROIs were (a) a position over the most significant cluster active during the stimulation phase and not active during the non-regulation phase and (b) being at the respective areas in the rACC and pInsL regions (15).

**Table 1:** MNI coordinates of the regions of interest used in the manuscript.

MNI coordinates			
ROI	x	y	z
rACC	−1	36	6
pInsL	−42	−20	9
pInsR	40	−13	6
MCC	−3	−25	38
aInsL	−45	6	−6
SII	−58	−29	23
UNR	−42	−61	29

rACC, rostral anterior cingulate cortex; pInsL, left posterior insula; pInsR, right posterior insula; SII, secondary somatosensory cortex; MCC, medial cingulate cortex; UNR, parieto-occipital area (control ROI).

The positioning of the UNR region was also monitored online during the training trials to not exhibit significant activation or deactivation. Four feedback conditions were used (see Table 2), assuming that for each target ROI the activity should be larger or smaller than the activity of the UNR region, displayed with up or down vertical directions in the ball displacement on the screen. Table 2 summarizes the four feedback conditions: ACCD, ACCU, INSD, INSU, the feedback signal computation (i.e., control ROI UNR), and the ball displacement on the screen.

**Table 2:** The four conditions for the generation of the feedback signal and their association with the ball displacement on the visual feedback.

Condition	Feedback	Ball displacement
1	ACCD: rACC—UNR < 0	Down
2	ACCU: rACC—UNR > 0	Up
3	INSD: pIns—UNR < 0	Down
4	INSU: pIns—UNR > 0	Up

(1) ACCD represents down-regulation of the BOLD activity in the rACC (compared with the UNR ROI). (2) ACCU represents up-regulation of the BOLD activity in the rACC (compared with the UNR ROI). (3) INSD represents down-regulation of the BOLD activity in the pINSL (compared with the UNR ROI). (4) INSU represents up-regulation of the BOLD activity in the INS (compared with the UNR ROI).

#### Offline fMRI data pre-processing and statistical analyses

The offline data pre-processing of the fMRI scans was performed using BrainVoyager QX 2.3 (Brain Innovation, Maastricht, The Netherlands, Goebel, 2001). Time courses for the brain connectivity analysis were extracted using an offline GLM analysis of all training fMRI datasets from six ROIs: the two target ROIs (rACC, pInsL), and four other ROIs selected for their involvement with pain processing: the medial cingulate gyrus (MCC), the right posterior insula (pInsR), the left anterior insula (aInsL), the left secondary somatosensory cortices (SIIL), see Figure 2 for their anatomical location.

Classification of Learners and non-Learners were based on the following criteria (14), see also Table 2:

- For conditions 1 and 2 (i.e., ACCD and ACCU), if the average difference of the activation of rACC and UNR was negative and positive respectively, and if this was the case for at least four out of six training trials, and if the modulation effect for the specific condition improved from trial 1 to trial 6, a subject was considered a Learner.
- For conditions 3 and 4 (i.e., INSD and INSU), if the average difference of the activation of pInsL and UNR was negative and positive respectively, and if this was the case for at least four out of six training trials, and if the modulation effect for the specific condition improved from trial 1 to trial 6, a subject was considered a Learner.

The other subjects were categorized as non-Learners.

### Functional connectivity analysis

**Figure 3:**
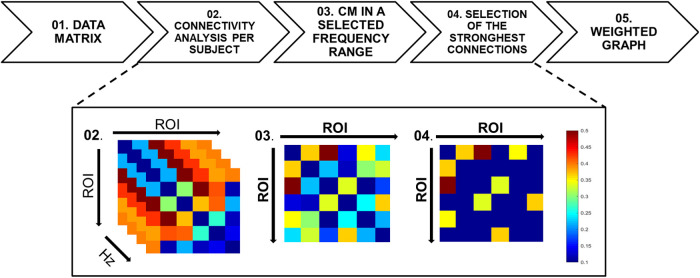
Flowchart of the functional connectivity analysis. (Top) The sequence of mathematical method used from the fMRI data matrix (step 1) to the generation of the weighted graphs (step 5). (Bottom) An example of fMRI data transformation from step 1 to 5. Step 1: for each participant, the fMRI data are represented by a matrix with dimensions *N* × *M*, in which the *N* dimension represents the ROIs and the *M* dimension represents number of samples of the time series (*N* = 6 and *M* = 258). Step 2: the coherence mathematical method is performed and a frequency-varying CM in the overall frequency range [0.0012–0.33] Hz is obtained with a frequency step of 0.0012 Hz. Step 3: After the average of the time-varying CM in the selected frequency range of interest [0.15–0.33] Hz, a single CM for each participant is obtained. For group analyses, the single subject CMs are first normalized in the range [0, 1], then averaged to obtain a group-level CM. Step 4: The CM is thresholded using the percentile thresholding method in order to enhance the strongest connections. Step 5: the CM is then used as an adjacency matrix for the graph extraction. The color bar indicates the connection strengths.

The Coherence function was used to evaluate the functional connectivity between the six ROIs for each of the four conditions (see Figure 3). The connectivity matrix (CM), which represents the level of inter- relationship between pairs of brain areas, was computed as a measure of the linear independence in the frequency domain between pairs of brain time-courses. Considering the six ROIs, a CM (6 × 6) was obtained and the values in the diagonal associated with the ROI self-similarity were set to zero. Based on the sampling rate (TR = 1.5 s), the frequency range investigated was [0.0012–0.33] Hz considering the limit due to the Nyquist theorem (*f_MAX_* = 2/*TR*). It was scanned with a frequency step of *f_step_* = *f_MIN_* = (1/*N*) *f_MAX_* = 0.0012 Hz where *N* = 258 is the number of samples acquired. The CMs for each subject for trials 1, 6 per condition were computed by averaging the overall frequency range. In the group analysis the connectivity matrices for the Learner and non-Learner groups were obtained by normalizing the CMs in the range [0, 1] and then averaging all of them by group. The normalization was necessary to make the results independent of subject variabilities of the CM levels. In the last step a cut-off threshold was established to extract the four strongest connections. Those connections were then plotted using a weighted graph representation for a visual inspection. In this graph the nodes are representative of the ROIs, and connections between ROIs pairs are for the CM weights above the defined threshold, otherwise the ROIs remain unconnected. Connectivity strengths were defined by weights of functional connectivity obtained from the coherence analysis. The colours code the strength of the connection between the ROIs. Colors from blue to red indicate weak to strong connection strength respectively and are expressed in arbitrary units (a.u).

In Figure 3 the analysis pipeline is shown as a flow chart representing the connectivity analysis from the data matrix to the CMs averaging in the frequency range and the threshold selection to evidence the strongest links. The simplest method for estimating functional connectivity in the frequency domain is Coherence analysis (35). The Coherence between two individual time-series (*y_i_*, *y_j_*) over a frequency range f is defined as follows:

$$Coh2i,j(f)=\frac{E[|(C_{i,j}(f){|}^{2}]}{E[\left|C_{i,i}(f)\right|]*E[\left|C_{\;j,j}(f)\right|]}$$

$$C_{i,j}(f)=\;Yi(f)Yj*(f)$$

The squared coefficient of Coherence can be interpreted as the proportion of the power in one of the two time-series (at a selected frequency), which can be explained by its linear relation with the other time course. Coherence is a positive function bounded by [0, 1] and symmetric in *i* and *j*. A measure of Coherency, such as an average over a frequency band, is capable of detecting zero-time lag synchronization and fixed time non-zero-time lag synchronization, which may occur when there is a significant delay between two brain sites. However, it does not provide any information on directionality of the coupling between the two recording sites.

**Figure 4:**
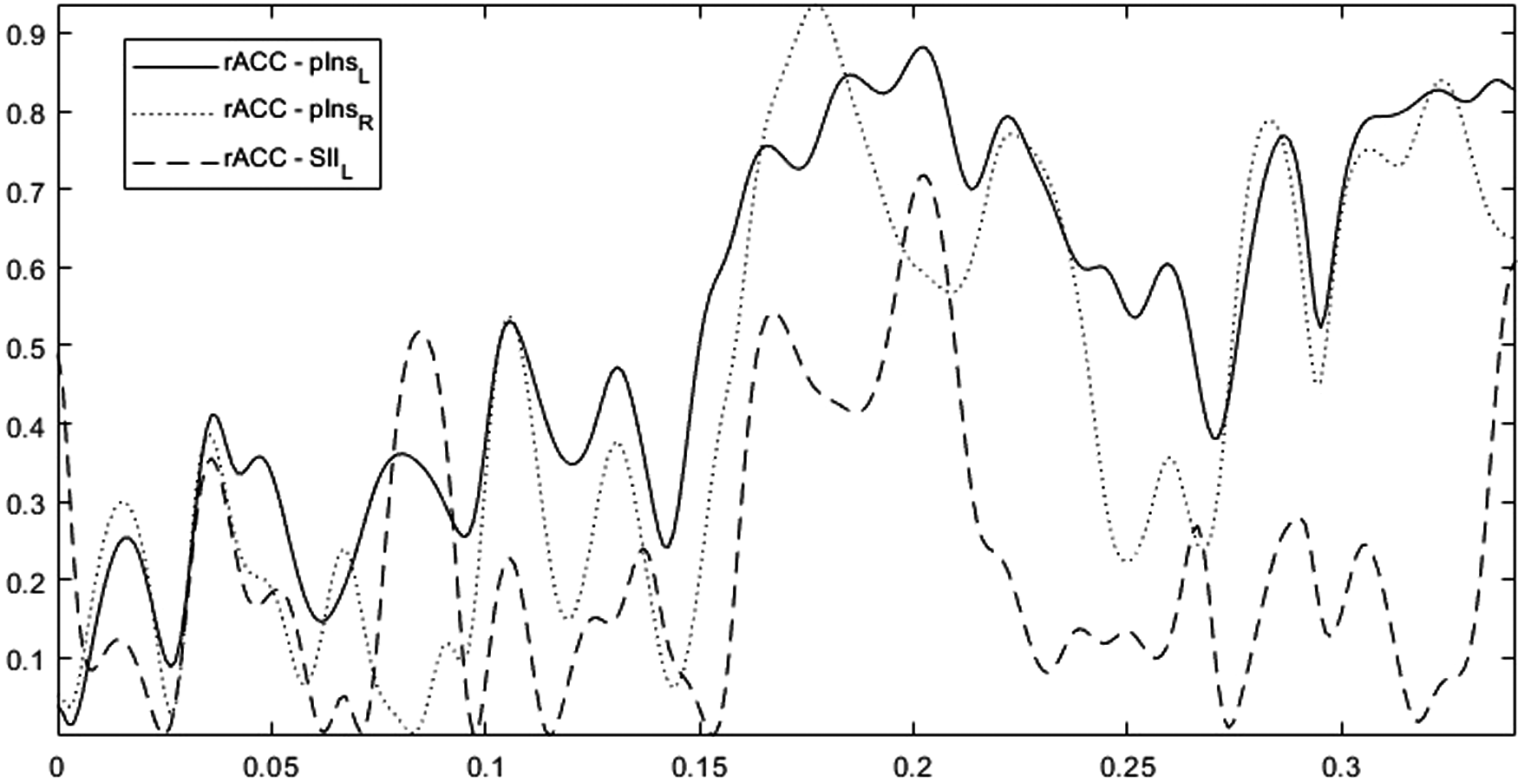
The trends of coherence versus frequency for the rACC paired with the ROIs pInsL, pInsR, SIIL for a representative learner (subject-4). The overall frequency range is [0.0012–0.33] Hz with a frequency step of 0.0012 Hz.

Figure 4 shows for a representative Learner (Subject-4), the trends of Coherence obtained for the ROI rACC paired with the ROIs [pInsL, pInsR, SIIL] versus frequency. We note a high variability in the frequency range. The analyses were then carried out averaging the information in the range [0.15–0.33] Hz. No change in the results was detected when changing this range. Due to the CM symmetry, we selected the four strongest connections over fifteen, the threshold value was set based on the 70th percentile and the CM was represented by a weighted and undirected graph.

In addition, we also assessed possible regulation of the control ROI, i.e., UNR ROI. We extracted time courses from the UNR ROI and calculated %BOLD signal change and compared it with rACC for both ACCD and ACCU conditions and with pInsL for both INSD and INSU conditions using paired t-tests (R package version 1.3.1093).

Finally, we compared pain intensity and unpleasantness ratings from the last training sessions (where the maximum effect would be expected) between Learners and non-Learners for all conditions using one way ANOVA (R package version 1.3.1093).

## Results

The functional connectivity analysis was investigated in the frequency domain at the group level and at a single subject level for the target ROIs in rACC and pInsL. The results are shown for the first trial (trial 1) and for the last trial (trial 6), the latter considered to be the trial when individuals had learned to regulate neural activity for ACC or Ins. The data showed high inter-subject variability in the learning outcome, which is not represented in the group results. We therefore show also individual results with the best and worst learning outcome in order to provide additional insights in the differences in connectivity. At the single subject level, we selected two participants, one representative of the Learner group (Subject-4) and the other one representative of the non-Learner group (Subject-8). The brain networks underlying the learning process were identified and compared among each other and with the group networks.

On average each condition includes four or five participants, with the exception of condition (INSD) in which the Learner and the non-Learner groups included seven and two participants, respectively (Table 3).

**Table 3:** Classification of participants in the learner or non-learner groups for each condition (ACCD, ACCU, INSD, INSU).

Condition	Learner	non-Learner group
ACCD	[3, 4, 5, 6, 7, 10]	[1, 2, 8, 9]
ACCU	[1, 2, 4, 6, 9]	[3, 5, 7, 8, 10]
INSD	[2, 3, 4, 5, 6, 8, 9, 10]	[1, 7]
INSU	[1, 2, 4, 6, 7]	[3, 5, 8, 9, 10]

Differences between Learners and non-Learners were found after normalization of the connectivity matrices in the range [0, 1] and thresholded using the percentile thresholding method to select the strongest connections. After such normalization and thresholding procedures, for ACCD, data from 4/6 Learners and 4/4 non-Learners remained.

**Figure 5:**
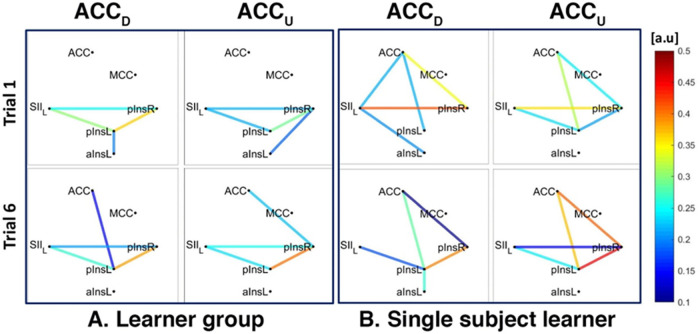
Connectivity strengths identified using the Coherence analysis for the ACC condition (ACC_D_, ACC_U_) in the Learner group (**A**) and for a representative Learner (**B**) in the first trial (Trial 1, top) and in the last trial (Trial 6, bottom). The colours from blue to red indicate weak to strong connection strength and are expressed in arbitrary units (a.u).

**Figure 6:**
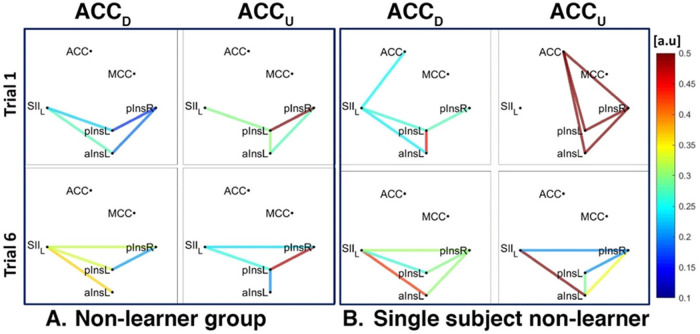
Connectivity strengths identified using the Coherence analysis for the ACC condition (ACC_D_, ACC_U_) in the non-Learner group (**A**) and for a representative non-Learner (**B**) in the first trial (Trial 1, top) and in the last trial (Trial 6, bottom). The colors from blue to red indicate weak to strong connection strength respectively and are expressed in arbitrary units (a.u).

For ACCD we found a connection between rACC and pInsL, mean connectivity indices between rACC and pInsL were (mean ± SD) 0.34 ± 0.25 for Learners and 0.30 ± 0.16 for non-Learners. For ACCU, data from 4/5 Learners and 5/5 non-Learners remained, and mean connectivity indices between rACC and pInsR were 0.61 ± 0.20 for Learners and 0.35 ± 0.19 for non-Learners (resp., Figures 5A, 6A).

In addition, for both ACCD and ACCU conditions and for both Learners and non-Learners, there was a network of three nodes, composed of SII, pInsL and pInsR that were interconnected at a group level (Figures 5A, 6A).

We also found a connection between left and right pIns for both ACCD and ACCU conditions for both Learners and non-Learners and for both group- and single-subject levels (Figures 5A,B, 6A,B).

In the non-Learner group for the ACCD and ACCU conditions, there was no connection between ACC and pInsL or pInsR, neither at a group level (Figure 6A) nor at a single subject level (Figure 6B). In addition, a connection between SII and aInsL is present for ACCD at a group and single subject level (Figure 6A left and Figure 6B left) and for ACCU at a single subject level (Figure 6B right).

**Figure 7:**
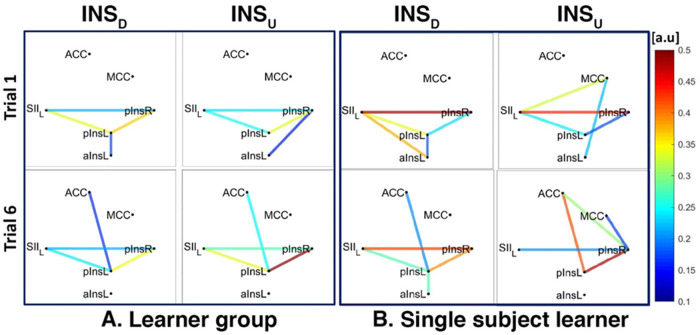
Connectivity strengths identified using the Coherence analysis for the Ins condition in the Learner group (**A**) and for a representative Learner (**B**) in the first trial (Trial 1, top) and in the last trial (Trial 6, bottom). The colors from blue to red indicate weak to strong connection strength respectively and are expressed in arbitrary units (a.u).

**Figure 8:**
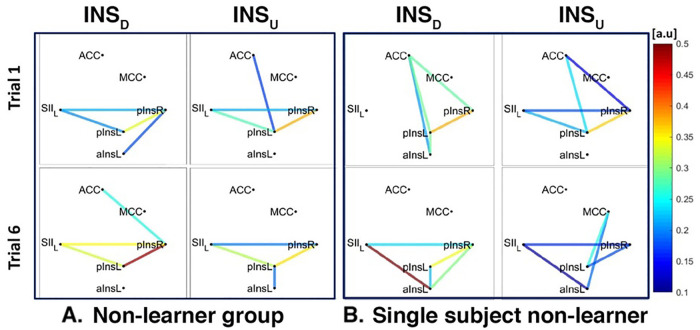
Connectivity strengths identified using the Coherence analysis for the Ins condition for the non-Learner group (**A**) and for a representative non-Learner (**B**) in the first trial (Trial 1, top) and in the last trial (Trial 6, bottom). The colors from blue to red indicate weak to strong connection strength respectively and are expressed in arbitrary units (a.u).

For INSD, after data normalization and thresholding, data from 7/8 Learners and 2/2 non-Learners remained. We found a connection between rACC and pInsL, mean connectivity indices between rACC and pInsL were (mean ± SD) 0.39 ± 0.20 for Learners and 0.23 ± 0.13 for non-Learners (resp., Figures 7A, 8A). In the non-Learners we found a connection between rACC and pInsR, mean connectivity indices between rACC and pInsR were 0.25 ± 0.08 for non-Learners and 0.14 ± 0.06 for non-Learners (resp., Figures 7A left, Figure 8A left).

For INSU, data from 4/5 Learners and 3/6 non-Learners remained. We found a connection between rACC and pInsL for the Learners, mean connectivity indices between rACC and pInsL were (mean ± SD) 0.29 ± 0.47 for Learners and 0.14 ± 0.11 for non-Learners (resp., Figures 7A, 8A).

The network of three interconnected nodes (SII, pInsL, pIns) was also found for INSD and INSU at a group level for both Learners and non-Learners (Figures 7A, 8A). This network was also found for the single subject Learner for INSD (Figure 7A left) but not for INSU and not for the single subject non-Learner (Figure 7B right, Figure 8B).

Regulation of the UNR ROI was significantly smaller compared with regulation of rACC for ACCD condition [*t*(1,9) = 5.62, *p* < 0.001], mean %BOLD signal change ± SD (UNR ROI: 0.03 ± 1.27 and rACC: 0.49 ± 1.79).

Regulation of the UNR ROI was significantly smaller compared with regulation of rACC for ACCU condition [*t*(1,9) = 5.00, *p* < 0.001], mean %BOLD signal change ± SD (UNR ROI: 0.05 ± 0.99 and rACC: 0.12 ± 2.35) for ACCU condition.

For INSD, regulation of the UNR ROI was significantly smaller compared with regulation of pInsL [*t*(1,9) = −3.29, *p* < 0.01], mean %BOLD signal change ± SD (UNR ROI: 0.05 ± 0.07 and pInsL: 0.26 ± 0.55).

For INSU, regulation of the UNR ROI was significantly smaller compared with regulation of pInsL [*t*(1,9) = −5.32, *p* < 0.001], mean %BOLD signal change ± SD (UNR ROI: 0.07 ± 0.10 and pInsL: 0.14 ± 0.17).

Finally, pain ratings did not differ significantly between Learners and non-Learners with respect to pain intensity and unpleasantness for all conditions [*F*(1,8) < 0.52, *p* > 0.50 for pain intensity and *F*(1,8) < 2.28, *p* > 0.17 for pain unpleasantness], Table 4.

**Table 4:** Pain intensity and unpleasantness ratings for all conditions (ACCD, ACCU, INSD, INSU) for learners (1) and non-learners (0) for trial 6.

Subjects	Condition	Learners (1)/non-Learners (0)	Pain intensity	Pain unpleasantness
S1	ACCD	0	5	7
S2	ACCD	0	5	5
S3	ACCD	1	6	6
S4	ACCD	1	5	6
S5	ACCD	1	7	7
S6	ACCD	1	5	5
S7	ACCD	1	5	5
S8	ACCD	0	4.5	7.5
S9	ACCD	0	7.5	7.5
S10	ACCD	1	7	6
S1	ACCU	1	7	10
S2	ACCU	1	5	5
S3	ACCU	0	5	6
S4	ACCU	1	4	4
S5	ACCU	0	6	6
S6	ACCU	1	6	7
S7	ACCU	0	6	5
S8	ACCU	0	2.5	5.5
S9	ACCU	1	5	5
S10	ACCU	0	5	6
S1	INSD	0	5	5
S2	INSD	1	4	4
S3	INSD	1	5	6
S4	INSD	1	5	5
S5	INSD	1	6	6
S6	INSD	1	4	6
S7	INSD	0	5	4
S8	INSD	1	4.5	7.5
S9	INSD	1	7	7
S10	INSD	1	5	4
S1	INSU	1	6	8
S2	INSU	1	5	5
S3	INSU	0	5	6
S4	INSU	1	5	6
S5	INSU	0	6	6
S6	INSU	1	4	5
S7	INSU	1	5	4
S8	INSU	0	3.5	6.5
S9	INSU	0	5	5
S10	INSU	0	9	9

The ratings relate to a verbal rating scale with pain intensity ranging from 0 = no pain to 10 = strongest imaginable pain and pain unpleasantness ranging from 0 = not unpleasant to 10 = extremely unpleasant. ACCD, rACC downregulation; ACCU, rACC upregulation; INSD, pInsL downregulation; INSU, pInsL upregulation.

## Discussion

We used a connectivity analysis framework in the frequency domain to examine up- or down-regulation of neural activity in the ACC and Ins and pain-associated brain areas during nociceptive processing. We found that successful up- and down-regulation of ACC and Ins is mediated by the ACC and by its functional connectivity with the posterior Insula. These findings are in line with the literature showing that individuals can learn to control activation in the ACC, a region known to be important for both pain perception and pain regulation (4, 12, 13, 36). We extended these findings by showing that we can not only down-regulate but also up-regulate neural activity in ACC and Ins. Such self-regulation aptitude could be related to factors such as pain coping (Emmert et al., 2017) and it would be interesting to investigate how pain coping relates to functional connectivity strength between SII, left and right posterior insula.

We also showed that voluntary control over activation in rACC and posterior Insula was consistently related to a network of three interconnected nodes composed of SII, left and right posterior insula. This network has been shown to be involved in pain processing (6, 37, 38), the nature of connections between the network nodes has, however, not been investigated. We found that the three nodes (SII, left and right posterior insula) were functionally connected, for both the Learner and the non-Learner group, although the strength of the connections differed between groups and conditions.

Interestingly, individuals who can successfully control activation in the Ins showed functional connections between ACC to pInsL (for both INSD and INSU). The non-Learner group showed an additional connection from ACC to the pInsR, albeit weaker. The non-Learner group showed connections between aInsL and SII (for ACCD), or between aInsL and pInsL (for ACCU and INSU). The Learner group did not show connections to aInsL. The anterior and posterior portions of the insula have been shown to be involved in different aspects of pain processing (38), with the posterior portion processing touch and pain sensation (37) and the anterior part involved in affective-motivational processes of pain perception (39). The connections to the aIns in the non-Learner group could therefore suggest that these individuals were negatively affected by pain perception, which might have disrupted their neural regulation task keeping attention on the nociceptive stimuli instead of regulation.

Despite successful control of the activation in ACC (down only) and pInsL (up and down) in the Learner group, pain intensity and unpleasantness ratings did not significantly differ between Learners and non-Learners. A lack of relationship between the regulation of brain activity and changes in behavior or cognition has also previously been reported (2, 17). A possible reason could be that the targets to be regulated, for example, ACC and pIns are involved in a variety of cognitive and behavioural functions such as emotional processing or somatosensory integration (17, 40–42) and may therefore not yield obvious specific behavioral or cognitive changes (43) and, in addition, pain as complex sensory and emotional experience may be more difficult to target by focusing of individual brain regions, as it involves many brain circuits (7).

### Limitations

This study involved four distinct conditions (ACCD, ACCU, INSD, INSU), over the course of 4 days. Switching between up- and down- regulation and between two different regions might have been challenging. This may be an additional reason why there were no significant differences in pain intensity and pain unpleasantness ratings between Learners and non-Learners.

Previous neurofeedback studies modulated only one target (4, 44) and used several training trials (up to four) on only 1 day. In this study, we increased the amount of training trials to six, giving subjects more time to train since two brain regions had to be upregulated and downregulated and the tasks were counterbalanced over the 4 days thus switching both the order of regions and the order of upregulation and downregulation. The use of separate training trials for the directions enabled to compare the course of controllability, as well as to monitor systematic changes that might occur independently from regulation efforts such as an overall decrease (habituation) or increase (sensitization) in the response of the area. Furthermore, we previously showed that the insula could be up and down regulated but ACC could only be down regulated, suggesting that both targets can be regulated successfully (apart from ACC upregulation). Moreover, we showed that success in the modulation of one region and direction of the modulation was not significantly correlated with success in another condition, indicating that regulation of one region might not interfere with regulation of another brain target (14).

## Conclusion

Real-time fMRI (rt-fMRI) enables self-regulation of neural activity in localised brain regions through neurofeedback. Previous studies showed successful up- and down-regulation of neural activity in the anterior cingulate cortex (ACC) and the insula (Ins) during nociceptive stimulation. In this work, the brain connectivity analysis was used to investigate how specific brain areas interact to enable successful regulation of nociceptive processing. A connectivity analysis framework in the frequency domain was used to identify a network of interconnected ROIs underlying regulation of neural activity during nociceptive processing. Both the analysis at group level and for single subjects showed that ACC is a key node for a successful control over somatosensory and pain-related areas, and pain regulation underlies an up-down control of ACC. Further work is needed to determine causal influences between somatosensory and pain-related areas during neural regulation of ACC and Ins.

## Data Availability

The raw data supporting the conclusions of this article will be made available by the authors, without undue reservation.
